# A new manganese-mediated, cobalt-catalyzed three-component synthesis of (diarylmethyl)sulfonamides

**DOI:** 10.3762/bjoc.10.39

**Published:** 2014-02-17

**Authors:** Antoine Pignon, Erwan Le Gall, Thierry Martens

**Affiliations:** 1 Électrochimie et Synthèse Organique, Institut de Chimie et des Matériaux Paris-Est, UMR 7182 CNRS, Université Paris-Est Créteil, 2-8 rue Henri Dunant, 94320 Thiais, France

**Keywords:** carbonyl compounds, cobalt, manganese, multicomponent reaction, organic bromides, sulfonamides

## Abstract

The synthesis of (diarylmethyl)sulfonamides and related compounds by a new manganese-mediated, cobalt-catalyzed three-component reaction between sulfonamides, carbonyl compounds and organic bromides is described. This organometallic Mannich-like process allows the formation of the coupling products within minutes at room temperature. A possible mechanism, emphasizing the crucial role of manganese is proposed.

## Introduction

(Diarylmethyl)amines constitute an important class of pharmacologically active compounds, displaying e.g. antihistaminic, antiarrhythmic, diuretic, antidepressive, laxative, anesthetic and anticholinergic activities [1–5]. These attractive properties have made them prominent synthetic targets for the organic chemist and various strategies, including for instance arylation of iminium salts [6], displacement of polymer-supported benzotriazole [7] with arylmagnesium reagents, addition of phenyllithium to selenoamides [8], addition of organometallic species [9–21] or arylboronic acids [22–27] to imines, reaction of organolithium and Grignard reagents with thioformamides [28], multistep reactions involving carbonyl compounds [29–30], intramolecular electrophilic arylation of lithiated ureas [31], and Petasis-type multicomponent reaction [32–34] have been reported for their preparation. Although these methods propose complementary approaches to the synthesis of (diarylmethyl)amines, their scope might be hampered by functional group incompatibilities or the number of steps. In addition, no straightforward and tunable method currently exists for the one-step preparation of (diarylmethyl)amines in N-protected form. Consequently, we felt that a multicomponent procedure intended to the synthesis of N-protected (diarymethyl)amines and related compounds would be highly desirable.

In preceding works, we described the Mannich-like multicomponent synthesis of α-branched amines from organic halides, aldehydes and amines, using a zinc-mediated process [35–38]. Although the scope of the reaction was demonstrated to be quite broad, some limitations were noticed, especially in reactions involving aryl halides. For instance, primary amines and enolizable aldehydes were unusable in this process. As a part of our ongoing efforts to improve reliability and versatility of multicomponent procedures, we describe herein a new manganese-mediated, cobalt-catalyzed three-component reaction, which circumvents the above-mentioned limitations by allowing the synthesis of an extended range of (diarylmethyl)sulfonamides (and related compounds) within minutes at room temperature. To the best of our knowledge, this constitutes the first manganese-mediated multicomponent reaction involving aryl halides as the source for nucleophilic species.

## Results and Discussion

We previously reported that primary amines are not active in a Mannich-like multicomponent reaction with aldehydes and arylzinc compounds [37]. It was assumed that the imine, which is initially formed by a condensation of the aldehyde and the amine, is not sufficiently electrophilic to undergo a coupling with the arylzinc compound. Considering the increased electrophilicity of sulfonylimines, we aimed to use sulfonamides as potential synthetic equivalents of ammonia in a procedure that additionally avoids the preformation of an imine and/or an organometallic compound. Thus, we turned our attention to a multicomponent reaction between aldehydes, aryl halides and sulfonamides, which involves an in situ activation (Barbier conditions) of the aryl halide by cobalt salts in the presence of a reducing metal. Initial experiments employed zinc as the reducing agent and were carried out under standard conditions. However, only trace amounts of the coupling product were detected in the reaction mixture. A screening of the reducing metal was then performed and revealed that only manganese is active in the process. Other reaction conditions such as the amount of reagents and catalyst, the solvent, the reaction time, the temperature and the nature of the halogen atom were also examined. The results are reported in Table 1.

**Table 1:** Optimization of the reaction conditions^a^.

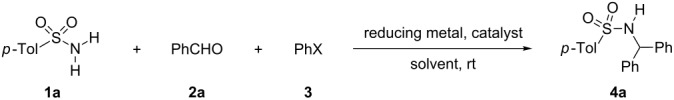								

Entry	PhX (equiv)^b^	X	Reducing metal	Amount (equiv)^c^	Cat. (mol %)^d^	Solvent	Time (min)	Yield (%)^e^

1	3	Br	Mg	10	–	THF	60	–
2	3	Br	Al	10	CoBr_2_ (15)	CH_3_CN	60	–
3	3	Br	Ti	10	CoBr_2_ (15)	CH_3_CN	60	–
4	3	Br	Cr	10	CoBr_2_ (15)	CH_3_CN	60	–
5	3	Br	Fe	10	CoBr_2_ (15)	CH_3_CN	60	–
6	3	Br	Sn	10	CoBr_2_ (15)	CH_3_CN	60	–
7	3	Br	Zn	10	CoBr_2_ (15)	CH_3_CN	60	–
**8**	**3**	**Br**	**Mn**	**10**	**CoBr_2_ (15)**	**CH_3_CN**	**5**	**68**
9	2.5	Br	Mn	10	CoBr_2_ (15)	CH_3_CN	5	52
10	2	Br	Mn	10	CoBr_2_ (15)	CH_3_CN	5	44
11	3	Br	Mn	15	CoBr_2_ (15)	CH_3_CN	5	76
12	3	Br	Mn	7.5	CoBr_2_ (15)	CH_3_CN	5	50
13	3	Br	Mn	5	CoBr_2_ (15)	CH_3_CN	5	32
14	3	Br	Mn	10	CoBr_2_ (15)	CH_3_CN	20	70
15	3	I	Mn	10	CoBr_2_ (15)	CH_3_CN	5	40
16	3	Cl	Mn	10	CoBr_2_ (15)	CH_3_CN	5	-
17	3	Br	Mn	10	CoBr_2_ (10)	CH_3_CN	5	52
18	3	Br	Mn	10	CoBr_2_ (1.5)	CH_3_CN	20	21
19	3	Br	Mn	10	–	CH_3_CN	60	–^f^
20	3	Br	Mn	10	NiBr_2_bpy (15)	CH_3_CN	60	–
21	3	Br	Mn	10	CoBr_2_ (15)	THF	60	–
22	3	Br	Mn	10	CoBr_2_ (15)	DMF	60	–
23	3	Br	Mn	10	CoBr_2_ (15)	Dioxane	60	–
24	3	Br	Mn	10	CoBr_2_ (15)	Toluene	60	–

^a^Reactions were conducted at room temperature with 5 mL of solvent, 0.43 g (2.5 mmol) of *p*-toluenesulfonamide (**1a**), 0.25 mL (2.5 mmol) of benzaldehyde (**2a**), 7.5 mmol of phenyl halide **3**, 1.125 mmol of the catalyst, and 25 mmol of the reducing metal, preactivated by using 0.1 mL BrCH_2_Ch_2_Br and 0.1 mL TFA. ^b^Calculated relative to the sulfonamide. ^c^Calculated relative to the sulfonamide. ^d^Calculated relative to the halide. ^e^GC yield. ^f^1 h at room temperature, then heating 5 h under reflux.

As mentioned above, metals other than manganese did not work in the reaction (Table 1, entries 1–7). In accordance with previously reported works, a minimum of two equiv of the halide were required for the reaction to take place. However, yields were noticeably improved by using 3 equiv of the halide (Table 1, entry 8) or 2.5 equiv (Table 1, entry 9) instead of 2 equiv (Table 1, entry 10). The amount of manganese was also of crucial importance. Indeed, whereas an increase to 15 equiv (Table 1, entry 11) led to a slightly improved yield, a decrease to 7.5 (Table 1, entry 12) or even 5 equiv (Table 1, entry 13) resulted in a dramatic decrease of the yield, which dropped to 32% in the latter case. However, the usage of an increased amount of manganese led to a more challenging work-up and the generation of more metallic waste. Consequently, for practical and environmental reasons, we chose to keep 10 equiv of manganese for the rest of the study. In general, the reaction proceeded very quickly and came to completion within 10 min at room temperature. An extended reaction time (20 min) did not result in a notably improved reaction yield (Table 1, entry 14).

Bromides proved to be the most efficient halides. Indeed, while the reaction also worked with iodides (Table 1, entry 15), albeit in lower yield, it did not work with chlorides (Table 1, entry 16). A decrease of the amount of cobalt salts to 10 mol % (Table 1, entry 17) or 1.5 mol % (Table 1, entry 18) resulted in a significant decrease of the reaction efficiency, thus indicating the prevalent role of the catalyst. This was confirmed by the absence of the coupling product when the reaction was conducted in the absence of cobalt bromide (Table 1, entry 19). Another nickel-based catalyst, e.g., NiBr_2_bpy, was not active under standard conditions (Table 1, entry 20), and solvents other than acetonitrile did not allow the reaction to proceed (Table 1, entries 21 to 24).

The scope of the reaction was then investigated by using various sulfonamides **1**, aldehydes **2** and organic bromides **3**, and the results are presented in Table 2.

**Table 2:** Scope of the reaction^a^.

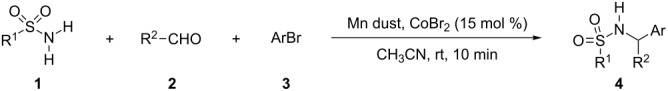						

Entry	R^1^	R^2^	Ar	Product		Yield (%)^b^

1	*p*-Tol	Ph	Ph	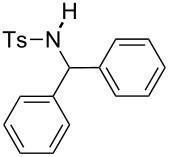	**4a**	68
2	*p*-Tol	4-MeO-C_6_H_4_	Ph	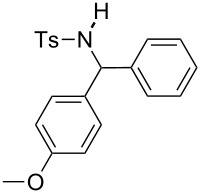	**4b**	60
3	*p*-Tol	3-thienyl	Ph	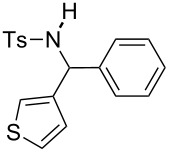	**4c**	15 (35^c^)
4	*p*-Tol	iPr	Ph	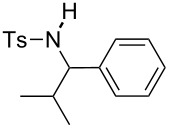	**4d**	45
5	*p*-Tol	Bn	Ph	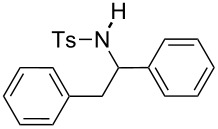	**4e**	32
6	*p*-Tol	Ph	4-Cl-C_6_H_4_	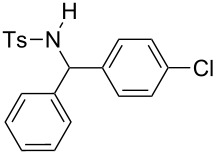	**4f**	39
7	*p*-Tol	Ph	3-CF_3_-C_6_H_4_	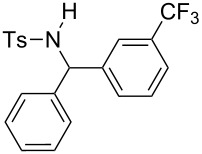	**4g**	32
8	*p*-Tol	Ph	4-EtO_2_C-C_6_H_4_	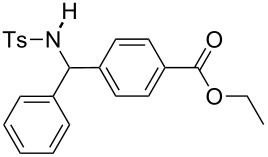	**4h**	46
9	*p*-Tol	Ph	4-iPr-C_6_H_4_	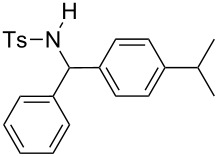	**4i**	20
10	*p*-Tol	4-Cl	3-Me-C_6_H_4_	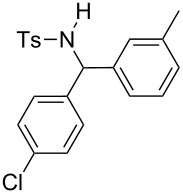	**4j**	25
11	*p*-Tol	4-F-C_6_H_4_	4-MeO-C_6_H_4_	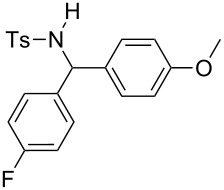	**4k**	34
12	Me	Ph	Ph	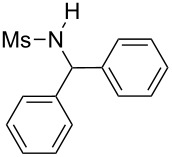	**4l**	65
13	Me	4-F-C_6_H_4_	Ph	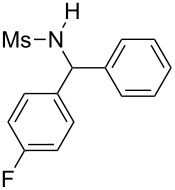	**4m**	27
14	Me	4-MeS-C_6_H_4_	Ph	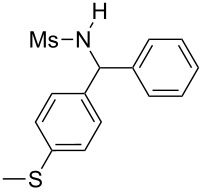	**4n**	36
15	Me	4-CF_3_-C_6_H_4_	4-Me-C_6_H_4_	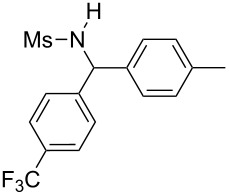	**4o**	39

^a^Reactions were conducted with 5 mL of acetonitrile, 2.5 mmol of sulfonamide **1**, 2.5 mmol of aldehyde **2**, 7.5 mmol of aryl bromide **3**, 0.25 g (1.125 mmol) of cobalt bromide, and 1.4 g (25 mmol) of manganese dust (preactivated by using 0.1 mL BrCH_2_Ch_2_Br and 0.1 mL TFA), for 10 min at room temperature. ^b^Isolated yield. ^c^Reaction conducted with the preformed sulfonyl imine.

Results indicated a rather broad functional tolerance of the reaction, although some yields are limited and might be likely improved under more specific conditions. In all cases, the organic bromide was completely consumed and the main side-products were the imine and the biaryl. The imine results from the reaction of the tosylamide with the aldehyde, and the biaryl was formed by reductive coupling of the starting halide. A preformed imine can be used in the process (Table 2, entry 3, result in brackets), indicating that this species might be the reactive electrophilic intermediate of the reaction. Aryl halide is generally prone to dimerization under such reductive conditions. Thus, we assume that if the imine is formed slowly or is less reactive, the more rapid consumption of the halide results in the lack of a nucleophilic partner in the reaction medium. In this case, the imine remains partly unconsumed at the end of the process. Nevertheless, we were pleased to observe that the reaction works with enolizable aldehydes (Table 2, entries 4 and 5), a notable result considering the acidity of the protons α to the carbonyl. Logically, Ms-containing sulfonamides (Table 2, entries 12–15) worked in the same fashion as their Ts-containing counterparts.

**Scheme 1:**
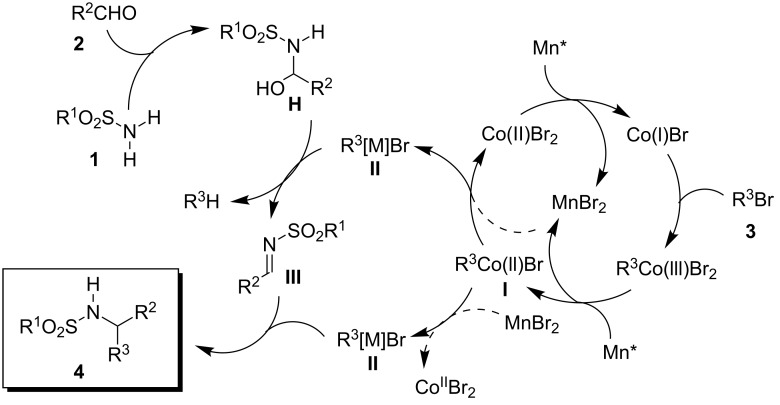
Possible reaction mechanism.

Considering the crucial importance of the reducing metal in the synthetic process and the probable involvement of an imine as the electrophilic intermediate, we were able to propose a possible reaction mechanism (Scheme 1).

It was previously established that zinc is able to reduce cobalt(II) in the presence of an aryl halide to promote the formation of a transient arylcobalt(II) species, which undergoes a fast transmetallation with zinc to furnish an organozinc species [39]. It can thus be assumed that a reductive metal other than zinc, e.g., manganese, can also promote a similar process. The first reaction step is the manganese-mediated formation of the organocobalt intermediate **I**. Depending on the kinetics of the transmetallation of **I** by manganese salts, two different scenarios may be envisaged featuring either an organocobalt or an organomanganese species as the key organometallic **II**.

In the first scenario, manganese undergoes a slow transmetallation with the organocobalt species **I**. It has been shown earlier that zinc salts undergo rapid transmetallations with organocobalt(II) species to furnish organozinc compounds. However, as mentioned above, the usage of zinc does not allow the reaction to proceed, so that we assume that the active organometallic species **II** could not be an organozinc compound. Provided manganese salts cannot undergo the transmetallation step at a comparable rate, but react significantly slower, it could be envisaged that the organometallic species **II** is in fact the organocobalt **I**. Thus, by transforming **I** into an inactive organozinc species, zinc salts would hamper the reaction.

In the other scenario, in which the transmetallation of **I** with manganese is very rapid, manganese plays a dual role by acting both as a reducing agent of cobalt (to form **I**) and as the active salt of a transmetallation step by forming an organomanganese reagent **II** (or a mixed manganese–cobalt-containing bimetallic compound). The intermediate **II** then acts both as a water scavenger at the stage of a formal hemiaminal intermediate **H** [40] to form the imine **III** and as a nucleophile furnishing **4** by trapping **III**.

## Conclusion

In conclusion, the results presented in this study indicate that the manganese(0)/cobalt(II) system is a suitable combination for the multicomponent synthesis of (diarylmethyl)sulfonamides and related compounds. Although this multicomponent reaction system has not been completely optimized yet, it is the first to allow the one-step preparation of (diarylmethyl)amines under a protected form. Current works include the examination of the effect of a manganese surface and granulometry on the reaction efficiency.

## Experimental

**General procedure:** A dried 50 mL round bottomed flask equipped with a reflux condenser was flushed with argon and charged with acetonitrile (5 mL). Manganese dust (1.4 g, 25 mmol), trifluoroacetic acid (0.1 mL, 1.3 mmol) and 1,2-dibromoethane (0.1 mL, 1.15 mmol) were added under vigorous (~500 rpm) stirring, and the mixture was heated to 60 °C. After cooling to room temperature, the organic bromide **3** (7.5 mmol), the aldehyde **2** (2.5 mmol), the sulfonamide **1** (2.5 mmol), and cobalt bromide (0.25 g, 1.125 mmol) were added successively, and the resulting mixture was stirred for 10 minutes at room temperature. The reaction mixture was poured into a sat. NH_4_Cl solution (75 mL) and extracted with diethyl ether (2 × 50 mL). The organic fractions were dried over Na_2_SO_4_ and concentrated under reduced pressure. The crude product was purified by silica-gel chromatography by using a solvent gradient (pentane/diethyl ether 90:10 to pentane/diethyl ether 70:30) to yield the three-component coupling product **4** as a generally white solid.
